# Supplementary material for: Structural insights into cobalamin loading and reactivation of human methionine synthase
Source: Nat Commun. 2026 May 11;17:6315. doi: 10.1038/s41467-026-72899-3 (PMC13376404; doi:10.1038/s41467-026-72899-3)
Supplement: Supplementary file 2 — Description of Additional Supplementary Files [file 41467_2026_72899_MOESM2_ESM.pdf]

Description of Additional Supplementary Files

Supplementary Movie 1: 3D variability analysis of apo C-half of MTR from the MeTHF dataset (Series 1). 3D variability analysis showing conformational heterogeneity in the apo C-half of MTR reconstruction.

Supplementary Movie 2: 3D variability analysis of apo C-half of MTR from the MeTHF dataset (Series 2). 3D variability analysis showing conformational heterogeneity in the apo C-half of MTR reconstruction.

Supplementary Movie 3: 3D variability analysis of HOCbl-bound C-half of MTR from the MeTHF, SAM, and HOCbl dataset (Series 1). 3D variability analysis showing conformational heterogeneity in the HOCbl-bound C-half of MTR reconstruction.

Supplementary Movie 4: 3D variability analysis of HOCbl-bound C-half of MTR from the MeTHF, SAM, and HOCbl dataset (Series 2). 3D variability analysis showing conformational heterogeneity in the HOCbl-bound C-half of MTR reconstruction.
