# Supplementary material for: Structural insights into cobalamin loading and reactivation of human methionine synthase
Source: Nat Commun. 2026 May 11;17:6315. doi: 10.1038/s41467-026-72899-3 (PMC13376404; doi:10.1038/s41467-026-72899-3)
Supplement: Supplementary file 7 — Reporting Summary [file 41467_2026_72899_MOESM7_ESM.pdf]

nature portfolio

Corresponding author(s): Wyatt W. Yue, wyatt.yue@newcastle.ac.uk
Thomas J. McCorvie,
thomas.mccorvie@newcastle.ac.uk

Last updated by author(s): Apr 26, 2026

# Reporting Summary

Nature Portfolio wishes to improve the reproducibility of the work that we publish. This form provides structure for consistency and transparency in reporting. For further information on Nature Portfolio policies, see our Editorial Policies and the Editorial Policy Checklist.

## Statistics

For all statistical analyses, confirm that the following items are present in the figure legend, table legend, main text, or Methods section.

| n/a | Confirmed | |
|---|---|---|
| ☐ | ☒ | The exact sample size ($n$) for each experimental group/condition, given as a discrete number and unit of measurement |
| ☐ | ☒ | A statement on whether measurements were taken from distinct samples or whether the same sample was measured repeatedly |
| ☒ | ☐ | The statistical test(s) used AND whether they are one- or two-sided<br>*Only common tests should be described solely by name; describe more complex techniques in the Methods section.* |
| ☒ | ☐ | A description of all covariates tested |
| ☒ | ☐ | A description of any assumptions or corrections, such as tests of normality and adjustment for multiple comparisons |
| ☐ | ☒ | A full description of the statistical parameters including central tendency (e.g. means) or other basic estimates (e.g. regression coefficient) AND variation (e.g. standard deviation) or associated estimates of uncertainty (e.g. confidence intervals) |
| ☒ | ☐ | For null hypothesis testing, the test statistic (e.g. $F$, $t$, $r$) with confidence intervals, effect sizes, degrees of freedom and $P$ value noted<br>*Give P values as exact values whenever suitable.* |
| ☒ | ☐ | For Bayesian analysis, information on the choice of priors and Markov chain Monte Carlo settings |
| ☒ | ☐ | For hierarchical and complex designs, identification of the appropriate level for tests and full reporting of outcomes |
| ☒ | ☐ | Estimates of effect sizes (e.g. Cohen's $d$, Pearson's $r$), indicating how they were calculated |

*Our web collection on statistics for biologists contains articles on many of the points above.*

## Software and code

Policy information about availability of computer code

| | |
|---|---|
| Data collection | DLS beamline Krios, York Structural Biology Laboratory Glacios, OmegaSTAR (BMG Biotech), QuantStudio 3 RT-PCR machine (Thermo Fisher Scientific |
| Data analysis | CryoSPARC-v4.2.1, GraphPad Prism v9, COOT v0.9.8.1, UCSF ChimeraX v1.5, PHENIX v1.20.1-4487, Isolde, AlphaFold3, Clustal Omega |

For manuscripts utilizing custom algorithms or software that are central to the research but not yet described in published literature, software must be made available to editors and reviewers. We strongly encourage code deposition in a community repository (e.g. GitHub). See the Nature Portfolio guidelines for submitting code & software for further information.

## Data

Policy information about availability of data

All manuscripts must include a data availability statement. This statement should provide the following information, where applicable:
- Accession codes, unique identifiers, or web links for publicly available datasets
- A description of any restrictions on data availability
- For clinical datasets or third party data, please ensure that the statement adheres to our policy

The authors declare that the main data supporting the findings of this study are available within the article and Supplementary Information. EM maps and models generated in this study of Human Methionine Synthase With Methyltetrahydrofolate, N-Half From Full-Length (EMDB-55190 [https://www.ebi.ac.uk/emdb/EMD-55190], PDB 9SSP [https://www.rcsb.org/structure/9SSP]), Human Methionine Synthase With Methyltetrahydrofolate, C-Half From Full-Length (EMDB-55191 [https://www.ebi.ac.uk/emdb/EMD-55191], PDB 9SSQ [https://www.rcsb.org/structure/9SSQ]), Human Methionine Synthase With Methyltetrahydrofolate,

Hydroxocobalamin, and SAM, N-Half From Full-Length (EMDB-55192 [https://www.ebi.ac.uk/emdb/EMD-55192], PDB 9SSR [https://www.rcsb.org/structure/9SSR]), Human Methionine Synthase With Methyltetrahydrofolate, Hydroxocobalamin, and SAM, C-Half His-ON From Full-Length (EMDB-55193 [https://www.ebi.ac.uk/emdb/EMD-55193], PDB 9SSS [https://www.rcsb.org/structure/9SSS]), Human Methionine Synthase With Methyltetrahydrofolate, Hydroxocobalamin, and SAM, C-Half His-OFF From Full-Length (EMDB-55194 [https://www.ebi.ac.uk/emdb/EMD-55194], PDB 9SST [https://www.rcsb.org/structure/9SST]), Human Methionine Synthase With Methylcobalamin, N-Half From Full-Length (EMDB-55195 [https://www.ebi.ac.uk/emdb/EMD-55195], PDB 9SSU [https://www.rcsb.org/structure/9SSU]) and Human Methionine Synthase With Methylcobalamin, Activation Domain From Full-Length (EMDB-55196 [https://www.ebi.ac.uk/emdb/EMD-55196], PDB 9SSV [https://www.rcsb.org/structure/9SSV]), have been deposited to the Electron Microscopy Data Bank (EMDB) and Protein Data Bank (PDB) Other structures referenced in this article are indicated, including PDB ID 4CCZ [https://www.rcsb.org/structure/4CCZ], 8G3H [https://www.rcsb.org/structure/8G3H], 8SSC [https://www.rcsb.org/structure/8SSC], 9CBO [https://www.rcsb.org/structure/9CBO], 9CBP [https://www.rcsb.org/structure/9CBP], 9CBQ [https://www.rcsb.org/structure/9CBQ], and 9CBR [https://www.rcsb.org/structure/9CBR]. Source data are provided with this paper.

## Human research participants

Policy information about studies involving human research participants and Sex and Gender in Research.

| | |
|---|---|
| Reporting on sex and gender | Not applicable - no group allocation was involved. |
| Population characteristics | Not applicable - no group allocation was involved. |
| Recruitment | Not applicable - no group allocation was involved. |
| Ethics oversight | Not applicable - no group allocation was involved. |

Note that full information on the approval of the study protocol must also be provided in the manuscript.

# Field-specific reporting

Please select the one below that is the best fit for your research. If you are not sure, read the appropriate sections before making your selection.

☒ Life sciences ☐ Behavioural & social sciences ☐ Ecological, evolutionary & environmental sciences

For a reference copy of the document with all sections, see nature.com/documents/nr-reporting-summary-flat.pdf

# Life sciences study design

All studies must disclose on these points even when the disclosure is negative.

| | |
|---|---|
| Sample size | The sample size determined by sufficient signal in experiment to ensure confidence in conclusions drawn from data. |
| Data exclusions | No data excluded. |
| Replication | Activity measurements were carried out in technical replicates of n = 2 or 3 as stated in figure legends |
| Randomization | Not applicable - no experimental groups were involved. |
| Blinding | Not applicable - no group allocation was involved. |

# Reporting for specific materials, systems and methods

We require information from authors about some types of materials, experimental systems and methods used in many studies. Here, indicate whether each material, system or method listed is relevant to your study. If you are not sure if a list item applies to your research, read the appropriate section before selecting a response.

## Materials & experimental systems

| n/a | Involved in the study | |
|---|---|---|
| ☒ | ☐ | Antibodies |
| ☐ | ☒ | Eukaryotic cell lines |
| ☒ | ☐ | Palaeontology and archaeology |
| ☒ | ☐ | Animals and other organisms |
| ☒ | ☐ | Clinical data |
| ☒ | ☐ | Dual use research of concern |

## Methods

| n/a | Involved in the study | |
|---|---|---|
| ☒ | ☐ | ChIP-seq |
| ☒ | ☐ | Flow cytometry |
| ☒ | ☐ | MRI-based neuroimaging |

## Eukaryotic cell lines

Policy information about cell lines and Sex and Gender in Research

| | |
|---|---|
| Cell line source(s) | Sf9 (Spodoptera frugiperda), #12659017, Gibco. |
| Authentication | No cells lines used were authenticated |
| Mycoplasma contamination | Cell lines were not tested for mycoplasma contamination |
| Commonly misidentified lines (See ICLAC register) | n/a |
