# Supplementary material for: Structural insights into cobalamin loading and reactivation of human methionine synthase
Source: Nat Commun. 2026 May 11;17:6315. doi: 10.1038/s41467-026-72899-3 (PMC13376404; doi:10.1038/s41467-026-72899-3)
Supplement: Supplementary file 8 — Transparent Peer Review file [file 41467_2026_72899_MOESM8_ESM.pdf]

nature portfolio

Peer Review File

Structural insights into cobalamin loading and reactivation of human methionine synthase

Corresponding Author: Dr Thomas McCorvie

This file contains all reviewer reports in order by version, followed by all author rebuttals in order by version.

Version 0:

Reviewer comments:

Reviewer #1

(Remarks to the Author)
This study provides structural insights into cobalamin, a highly important and rare cofactor, and the subsequent reactivation of the human methionine synthase enzyme, which plays a crucial role in several rare inherited metabolic disorders. Overall, this is a very strong technical paper that combines cryo-EM, biochemistry, and AlphaFold to investigate the interaction of MTR with its cofactor and partner proteins. The structural insights and conclusions drawn from this impressive study are convincing, particularly the use of structural snapshots to elucidate interactions with partner proteins. The manuscript is well written, logically structured, and experimentally thorough, and the conclusions are largely supported by the data. However, a few concerns should be addressed.

A central question concerns the physiological interpretation of the cryo-EM structures, given that the full-length MTR protein is not observed directly and instead the N-terminal and C-terminal halves are resolved independently. It would be helpful if the authors could expand the discussion on why this behavior is observed and how it reflects the native dynamics of the enzyme rather than effects related to sample preparation, surface interactions, or partial dissociation. In this context, clarifying what the final peak in the size-exclusion chromatogram represents would also help readers better understand the biochemical state of the sample used for structural analysis.

Related to the point above, a brief description of the strategies explored to stabilize full-length MTR for cryo-EM—such as the use of mild detergents, cross-linkers, or alternative grid supports would be valuable. Even if these approaches were unsuccessful, describing them would provide useful context and strengthen confidence in the robustness of the conclusions drawn from the observed states.

The identification and interpretation of His-on and His-off states of the cobalamin-bound enzyme are particularly interesting. It would be useful to clarify whether focused classification or focused refinement approaches were applied to enhance local resolution around the cobalamin and the coordinating histidine residue, and whether combining such approaches with three-dimensional variability analysis might further refine the description of the conformational landscape.

When assessing cobalamin occupancy based on absorbance at 350 nm, it may be helpful to support this measurement with an orthogonal quantitative method such as ICP–MS, especially given that reducing agents like TCEP can sometimes influence optical readouts. Such complementary measurements would further reinforce the conclusions regarding cofactor binding and loading.

Were there any efforts to solve structures of MTRR or MMADHC in complex with MTR? AlphaFold3 predictions may not be fully relevant if the observed MTR remains as independent halves. While complementary interaction assays are presented, structural characterization would provide a clearer picture of these interactions and enable more confident interpretation of residue-level contacts, which are currently difficult to infer from the provided data.

Minor comments:

The approximately 1.16 kDa difference between the theoretical and measured molecular weights observed by native mass spectrometry should be clarified.

Cleaning the cryo-EM maps to remove dust or spurious densities would improve figure clarity.

In Fig. 2a, showing the local density around the coordinating histidine residue would help readers visually assess the basis for assigning the His-on and His-off states.

In summary, this is a strong and impactful contribution that provides valuable new insights into the structural dynamics and regulation of human methionine synthase. Addressing the points above would further strengthen the manuscript and clarify the physiological relevance and mechanistic implications of the findings.

Reviewer #2

(Remarks to the Author)
The manuscript combines cryo-EM, AlphaFold3 modeling, and biochemical assays (including pull-down experiments and enzymatic activity measurements) to develop mechanistic models for MTR modularity, cobalamin loading, and reductive reactivation. Overall, the approach is well conceived, and the individual datasets are of good quality. That said, several of the central mechanistic conclusions rest heavily on structural inference and predictive modeling. In my view, the manuscript would be stronger with either additional experimental support or more restrained framing of some of these interpretations. In addition, the eukaryote-specific regulatory features of the system could be highlighted more clearly, as this is an important distinction from bacterial MetH systems and a strength of the study.

In particular, a number of the proposed interaction architectures—most notably those involving MTR–MMADHC and MTR–MTRR assemblies—are supported primarily by AlphaFold3 models and qualitative binding data. AlphaFold3 is a valuable hypothesis-generating tool, but by itself it does not establish that a given interface forms in solution or plays a functional role during catalysis or reactivation. This limitation is especially relevant for the newly proposed non-canonical interfaces between the MTR C-terminal region and regions of MTRR outside the well-established FMN–Act interaction.

Related to this point, the manuscript interprets AF3 models of MTRR–MTR complexes in ways that involve the FMN-, FAD-, and NADPH-binding regions of MTRR. Because the AlphaFold3 server does not explicitly model small-molecule cofactors such as FMN or NADPH, it would be helpful for the authors to clarify how ligand context was handled in the modeling. Were the AF3 models generated without ligands, with cofactors subsequently placed by superposition from experimental structures or homologous reductases, or are interactions involving these regions inferred solely from protein geometry? In addition, clarification of the assumed oxidation states of these cofactors would be helpful. Making these assumptions explicit would allow readers to better judge how strongly the proposed interfaces and domain orientations depend on ligand-bound states.

More broadly, several of the key ideas in the manuscript—particularly the proposed modular behavior of MTR and ligand-dependent rearrangements across functional states—would benefit from solution-phase experimental restraints that directly report on interdomain and interprotein contacts. In this regard, cross-linking mass spectrometry (XL-MS) seems particularly well suited to the system. Even a relatively limited XL-MS dataset could help validate the proposed MTR–MMADHC and MTR–MTRR interfaces and test whether domain proximities change across different ligand conditions. The system also appears amenable to comparative or semi-quantitative XL-MS approaches. Comparing cross-link patterns across apo MTR, substrate-bound states (e.g., MeTHF/SAM), HOCbl-bound states, and MeCbl-bound states would provide more direct evidence for ligand-dependent rearrangements than can be inferred from separate structural snapshots alone. Such data would substantially strengthen the link between the structural models and the enzymatic cycle. These experiments need not be exhaustive to be informative; a focused analysis using a single cross-linker and a small number of carefully chosen conditions would already add significant weight. If additional experiments are not feasible, I would encourage the authors to more clearly present the AF3-derived architectures as working models rather than definitive mechanistic intermediates.

The manuscript also makes several assignments regarding cobalamin coordination and oxidation state, particularly in distinguishing His-on versus His-off HOCbl-bound states. While the structural distinctions are convincing, the oxidation state of cobalt is inferred rather than directly measured. Given the central role of cobalt redox chemistry in MTR catalysis and reactivation, complementary spectroscopic evidence (such as UV–Vis and/or EPR) obtained on the same samples used for structural analysis would substantially strengthen these assignments. If such data are not available, the corresponding language should be framed more cautiously.

Finally, the overall study design would benefit from clearer presentation in the context of the catalytic cycle and the experimentally observed bound states in the early section of the manuscript. In particular, it is not immediately clear how the EM structure shown in Figure 2 maps onto two cobalt oxidation states summarized in Figure 7. The authors may wish to consider adding a schematic figure that explicitly links each structural snapshot (e.g., MeTHF, OHCbl, MeCbl) to its corresponding position in the catalytic and reactivation cycles, including the associated ligand and oxidation states. Such a figure would help readers more easily follow how the cryo-EM structures, biochemical assays, and proposed mechanistic model fit together.

Other comments: editorial corrections and clarifications

Page 2, line 84
“MMACHC” should be corrected to MTRR.

Page 2, line 90

The abbreviation “Conn” may not be standard terminology in the literature. Please consider replacing it with a more descriptive term.

Page 18, line ~21
“Effect” should be replaced with “affect” where the verb form is intended.

Page 16, line ~582 (“…explains its long hinge region”)
The wording could be read as implying a mechanistic explanation without clear context. Please elaborate and clarify whether the data demonstrate or are merely consistent with the proposed mechanism.

Page 16, line ~592 (“…to the lower sequence conservation”)
Please clarify whether the stated differences arise from specific interacting residues, linker length, or a combination of both.

Page 17, Figure 7
Panel labeling appears inconsistent: “(i) Reactivation Cycle” should be “(ii) Reactivation Cycle.”

Page 18, line 655 (“insights into eukaryote-unique aspects”)
This sentence would benefit from a more concise summary of the eukaryote-specific features highlighted by the study.

Reviewer #3

(Remarks to the Author)
I co-reviewed this manuscript with one of the reviewers who provided the listed reports. This is part of the Nature Communications initiative to facilitate training in peer review and to provide appropriate recognition for Early Career Researchers who co-review manuscripts.

Version 1:

Reviewer comments:

Reviewer #1

(Remarks to the Author)
The authors have adequately answered all of my concerns. The changes to the discussion and the figures provide excellent clarifications to my original queries.

Reviewer #2

(Remarks to the Author)
The authors have addressed my comments; the manuscript is ready for acceptance.

Reviewer #3

(Remarks to the Author)
I co-reviewed this manuscript with one of the reviewers who provided the listed reports. This is part of the Nature Communications initiative to facilitate training in peer review and to provide appropriate recognition for Early Career Researchers who co-review manuscripts.

Open Access This Peer Review File is licensed under a Creative Commons Attribution 4.0 International License, which permits use, sharing, adaptation, distribution and reproduction in any medium or format, as long as you give appropriate credit to the original author(s) and the source, provide a link to the Creative Commons license, and indicate if changes were made.
In cases where reviewers are anonymous, credit should be given to 'Anonymous Referee' and the source.
The images or other third party material in this Peer Review File are included in the article's Creative Commons license, unless indicated otherwise in a credit line to the material. If material is not included in the article's Creative Commons license and your intended use is not permitted by statutory regulation or exceeds the permitted use, you will need to obtain permission directly from the copyright holder.
To view a copy of this license, visit https://creativecommons.org/licenses/by/4.0/

REVIEWER COMMENTS

Reviewer #1 (Remarks to the Author):

This study provides structural insights into cobalamin, a highly important and rare cofactor, and the subsequent reactivation of the human methionine synthase enzyme, which plays a crucial role in several rare inherited metabolic disorders. Overall, this is a very strong technical paper that combines cryo-EM, biochemistry, and AlphaFold to investigate the interaction of MTR with its cofactor and partner proteins. The structural insights and conclusions drawn from this impressive study are convincing, particularly the use of structural snapshots to elucidate interactions with partner proteins. The manuscript is well written, logically structured, and experimentally thorough, and the conclusions are largely supported by the data. However, a few concerns should be addressed.

A central question concerns the physiological interpretation of the cryo-EM structures, given that the full-length MTR protein is not observed directly and instead the N-terminal and C-terminal halves are resolved independently. It would be helpful if the authors could expand the discussion on why this behavior is observed and how it reflects the native dynamics of the enzyme rather than effects related to sample preparation, surface interactions, or partial dissociation. In this context, clarifying what the final peak in the size-exclusion chromatogram represents would also help readers better understand the biochemical state of the sample used for structural analysis.

Response- Thank you for this comment. We agree that the discussion would benefit from relating the native dynamics of MTR to our cryo-EM data, where MTR was observed as two independent halves.

From previous studies on the MTR homologue MetH it is widely understood that during each of MTR's three methylation reactions, it undergoes large conformational changes, with multiple domains interacting with the Cob during the catalytic and reactivation cycles. In human MTR, this flexibility is enabled by four linkers connecting the different domains, as shown in Figure 1a, including a rigid linker (Linker 1) connecting Hcy and Fol domains, and a notably flexible linker connecting the N- and C- halves of MTR (Linker 2).

The reactivation conformation of MTR has been characterised by the interaction of all three domains of C-half MTR (Cap, Cob, and Act), forming a rigid module of C-half MTR, while the N-half remains rigid given the Linker 1. We interpret that the independent reconstruction of each half of MTR from the full-length protein is due to its conformational flexibility, limiting the alignment of full-length particles and favouring the classification around the two rigid modules present in the sample. This behaviour is also consistent with the data obtained from MeCbl-bound MTR, in which the protein conformation allows the formation of three independent bodies (connected by Linkers 2 and 4).

We have expanded the Discussion section to better interpret the data related to MTR's native dynamics. Importantly, the cryo-EM samples were prepared from the monomeric MTR fraction, analysed by biochemical methods (SDS-PAGE and native mass spectrometry), which supports that the sample corresponds to the apo full-length MTR.

Regarding the size-exclusion performed and shown in Supplementary Fig. 1a, it was performed with the elution of MTR purification from nickel affinity column. Contaminants may still be present, thus besides separating the two different states of MTR (aggregated and monomeric), it also separates low molecular weight contaminants. We have updated the figure and legend, labelling the final peak accordingly (Contaminant Proteins).

In the first paragraph of discussion, we have added:

Line 514-525 "In our datasets, these modules adopt a broad distribution of relative orientations, supporting the enzyme's capability of carrying out multiple methylation reactions. This nonetheless has imposed a technical constraint during cryo-EM processing, preventing the recovery of 2D classes representing the full-length protein. Importantly, we reasoned that the 2D classes did not represent truncated MTR proteins, because all grids were prepared using the monomeric peak from size-exclusion chromatography, which represents apo full-length MTR based on analyses prior to vitrification. Therefore, we attribute MTR appearing as separated modules in cryo-EM primarily to its dynamic nature and flexible linkers, resulting in conformational heterogeneity. While we cannot exclude factors such as air-water interface effects, and the use of mild detergents or carbon support grids could potentially yield classes of full-length protein, we reason that the modular behaviour found is more consistent with MTR dynamics and flexibility."

Related to the point above, a brief description of the strategies explored to stabilize full-length MTR for cryo-EM—such as the use of mild detergents, cross-linkers, or alternative grid supports would be valuable. Even if these approaches were unsuccessful, describing them would provide useful context and strengthen confidence in the robustness of the conclusions drawn from the observed states.

Response- Regarding attempted strategies to stabilize full-length MTR for cryo-EM we have initially explored the use of two different grid types, namely holey carbon-supported gold and UltrAuFoil grids, where we found that we got more and better 2D classes of the C-half with the pure gold grids. This was likely due to having thinner ice and less movement as has been reported for UltrAuFoil grids. We additionally employed the substrate methyltetrahydrofolate based off our observations that it thermally stabilised the full-length apo protein. It is our opinion that without prior structural evidence of a specific arrangement without cross-linkers that they should be avoided as they may induce artefacts. On the other hand, detergents or carbon/graphene oxide support may reduce any denaturation due to air-water interface affects (though may cause orientation bias) and is something that we have planned for future experiments.

We have now briefly expanded on what strategies we have used:

Line 140-141: "Initial screening using holey carbon support grids gave no discernible 2D classes likely due to ice thickness. However, switching to pure gold UltrAuFoil grids……."

Line 522-524: "While we cannot exclude factors such as air-water interface effects, and the use of mild detergents or carbon support grids could potentially yield classes of full-length protein…"

The identification and interpretation of His-on and His-off states of the cobalamin-bound enzyme are particularly interesting. It would be useful to clarify whether focused classification or focused refinement approaches were applied to enhance local resolution around the cobalamin and the coordinating histidine residue, and whether combining such approaches with three-dimensional variability analysis might further refine the description of the conformational landscape.

Response- We did attempt focused refinement and focused classification around the cobalamin and SAM binding site in our His-on and His-off state maps. Unfortunately, they did not result in any appreciable difference or increased quality for these maps, and no other states could be determined.

We have added a sentence explaining this in the Methods section:

Line 889-892: "Further attempts of masked local refinement and masked 3D classification around the cobalamin and SAM binding sites for these states did not result in any appreciable improvement in map quality nor identified any further states."

When assessing cobalamin occupancy based on absorbance at 350 nm, it may be helpful to support this measurement with an orthogonal quantitative method such as ICP–MS, especially given that reducing agents like TCEP can sometimes influence optical readouts. Such complementary measurements would further reinforce the conclusions regarding cofactor binding and loading.

Response- We agree that a quantitative method such as ICP-MS would provide an extra, independent measure of Cbl occupancy and strengthen our findings using SEC. In this study, we analysed Cbl loading to MTR and MMADHC-dependent loading through SEC-based readout by monitoring absorbance at 350 nm. Importantly, the buffer used for the assays did not contain any TCEP, and any traces of it would be from the purification process and were further diluted prior to the experiments. Therefore, we consider it is unlikely that TCEP had a substantial role in the observed 350 nm signal in the assay.

We have not performed extra experiments such as ICP-MS because they are beyond the scope of our current study focused on structural characterisation. The SEC-based experiments were performed in duplicate, and showed a consistent behaviour, which supports our conclusions on Cbl binding/delivery. We note that ICP-MS would strength further our conclusions and could be pursued on future work depending on protein availability and facility access.

Were there any efforts to solve structures of MTRR or MMADHC in complex with MTR? AlphaFold3 predictions may not be fully relevant if the observed MTR remains as independent halves. While complementary interaction assays are presented, structural characterization would provide a clearer picture of these interactions and enable more confident interpretation of residue-level contacts, which are currently difficult to infer from the provided data.

Response- We agree that structural data on the predicted complexes (MTR-MTRR and MTR-MMADHC) would increase confidence and reveal residue-level details of these interactions. Although we understand the MTR flexibility might be a challenge

for cryo-EM work on these complexes, we expect that both MTRR and MMADHC bind MTR through its C-terminal half, especially when considering MTR in the reactivation conformation.

Efforts were made to obtain cryo-EM data on MTR-MTRR using the complex composed of the full-length proteins. However, due to the flexibility of both proteins, the data did not yield interpretable 2D class averages. Therefore, we concluded that rounds of sample and grid optimisation would be needed to achieve a better dataset, and hence this work suit better in future follow-on manuscripts. Equivalent analysis using full-length MTR-MMADHC has not been pursued.

To address the flexibility issue, we are currently working on sample optimization for cryo-EM using the truncated constructs shown here to interact in the pulldown assays (MTR, MTRR, and MMADHC). These constructs, each composed of the interaction regions, are less flexible and should be more suitable to reveal details on the protein-protein interacting regions, although they require further optimisation. Finally, we also understand that the AF3 predictions were used as a starting point to generate hypotheses by proposing possible interaction regions between these proteins, which were assessed by pulldown assays using truncated constructs. Structural studies and additional biophysical assays would refine the molecular basis of these interactions and support in-depth analysis of the effects of ligands on complex formation which we believe are beyond the scope of our current manuscript.

Minor comments:

The approximately 1.16 kDa difference between the theoretical and measured molecular weights observed by native mass spectrometry should be clarified.

Response- Thank you for pointing this out. There was an error, and the calculated molecular mass of our intact MTR^{FL} sample should be 142.38 kDa, which is 0.3 kDa lower than the mass determined by native mass spectrometry. The error occurred because the initial mass was calculated for the full-length MTR (residues 1-1265), but the construct used in this study is a near full-length protein, comprising residues 16-1265, in which the initial 15 residues were truncated since they are predicted to be disordered. We have now specified this in the Methods section.

The remaining determined mass difference of ~0.3 kDa is consistent with post-translational modifications introduced during protein expression in Sf9 cells. However, we did not further assess PTMs in the sample in this study. An alternative explanation for the difference could reflect uncertainty in the mass spectrometry, since it represents roughly 0.2% of difference in mass.

Cleaning the cryo-EM maps to remove dust or spurious densities would improve figure clarity.

Response- We have updated the figures containing cryo-EM map images with the Hide Dust function in ChimeraX to improve figure clarity.

In Fig. 2a, showing the local density around the coordinating histidine residue would help readers visually assess the basis for assigning the His-on and His-off states.

Response- Thank you for the suggestion. We believe that the reviewer is referring to Fig 2c which is a zoom-in of H785 and the cobalamin in our His-off and His-on structures. We have updated both panels to show the density around the histidine residue.

In summary, this is a strong and impactful contribution that provides valuable new insights into the structural dynamics and regulation of human methionine synthase. Addressing the points above would further strengthen the manuscript and clarify the physiological relevance and mechanistic implications of the findings.

Response- Thank you for your review and comments on our submitted manuscript.

Reviewer #2 (Remarks to the Author):

The manuscript combines cryo-EM, AlphaFold3 modeling, and biochemical assays (including pull-down experiments and enzymatic activity measurements) to develop mechanistic models for MTR modularity, cobalamin loading, and reductive reactivation. Overall, the approach is well conceived, and the individual datasets are of good quality. That said, several of the central mechanistic conclusions rest heavily on structural inference and predictive modeling. In my view, the manuscript would be stronger with either additional experimental support or more restrained framing of some of these interpretations. In addition, the eukaryote-specific regulatory features of the system could be highlighted more clearly, as this is an important distinction from bacterial MetH systems and a strength of the study.

In particular, a number of the proposed interaction architectures—most notably those involving MTR–MMADHC and MTR–MTRR assemblies—are supported primarily by AlphaFold3 models and qualitative binding data. AlphaFold3 is a valuable hypothesis-generating tool, but by itself it does not establish that a given interface forms in solution or plays a functional role during catalysis or reactivation. This limitation is especially relevant for the newly proposed non-canonical interfaces between the MTR C-terminal region and regions of MTRR outside the well-established FMN–Act interaction.

Related to this point, the manuscript interprets AF3 models of MTRR–MTR complexes in ways that involve the FMN-, FAD-, and NADPH-binding regions of MTRR. Because the AlphaFold3 server does not explicitly model small-molecule cofactors such as FMN or NADPH, it would be helpful for the authors to clarify how ligand context was handled in the modeling. Were the AF3 models generated without ligands, with cofactors subsequently placed by superposition from experimental structures or homologous reductases, or are interactions involving these regions inferred solely from protein geometry? In addition, clarification of the assumed oxidation states of these cofactors would be helpful. Making these assumptions explicit would allow readers to better judge how strongly the proposed interfaces and domain orientations depend on ligand-bound states.

Response- Thank you for the comments. We agree that the AF3 predictions are a hypothesis-generating tool, to prompt further investigation using biophysical and structural work. Although pulldown assays using truncated constructs support the predicted interactions and the protein regions involved, we are aware that the exact interacting interface cannot be fully revealed with certainty by AF3 predictions alone. We have now clarified this point in the discussion.

Line 608-610: "Although the novel interaction could be confirmed using qualitative biophysical assays, further structural characterisation would help define the interaction interface."

The complexes were predicted without the addition of any ligands or cofactors (we have now clarified this further in the Methods section as below.

Line 919-920: "All predictions were performed without the addition of ligands or cofactors".

But in some instances, where specified, the AF3-predicted models were superimposed onto structures from experimental work containing ligands or cofactors (e.g., Fig. 4b, in which the modelled MMADHC–MTR complex was superimposed onto the cryo-EM structure of the His-off C-half of MTR). This superimposition was performed purely to assess whether the predicted complex is compatible with the experimental structures and whether any steric clashes would be observed (none were observed).

Furthermore, all the biophysical assays were performed on samples without added ligands, except for the FAD and FMN bound to MTRR during recombinant expression. Both these flavins are oxidised in our samples, as evidenced by the yellow colour of the recombinant proteins and no attempts were made to reduce them. Additionally, AF3 currently cannot perform predictions for different oxidation states. As there are multiple combinations of ligands and oxidation states that could be addressed, we believe that studying the influence of ligands on complex formation would be better addressed in future studies involving biophysical, spectrophotometric and structural analysis of these complexes.

More broadly, several of the key ideas in the manuscript—particularly the proposed modular behavior of MTR and ligand-dependent rearrangements across functional states—would benefit from solution-phase experimental restraints that directly report on interdomain and interprotein contacts. In this regard, cross-linking mass spectrometry (XL-MS) seems particularly well suited to the system. Even a relatively limited XL-MS dataset could help validate the proposed MTR–MMADHC and MTR–MTRR interfaces and test whether domain proximities change across different ligand conditions. The system also appears amenable to comparative or semi-quantitative XL-MS approaches. Comparing cross-link patterns across apo MTR, substrate-bound states (e.g., MeTHF/SAM), HOCbl-bound states, and MeCbl-bound states would provide more direct evidence for ligand-dependent rearrangements than can be inferred from separate structural snapshots alone. Such data would substantially strengthen the link between the structural models and the enzymatic cycle. These experiments need not be exhaustive to be informative; a focused analysis using a single cross-linker and a small number of carefully chosen conditions would already add significant weight. If additional experiments are not feasible, I would encourage the authors to more clearly present the AF3-derived architectures as working models rather than definitive mechanistic intermediates.

Response- Thank you for the suggestion on the XL-MS. We agree that further work is required to confirm the various domain arrangements of MTR in its different states and importantly to confirm the interfaces of its predicted complexes with MMADHC and MTRR. However, we believe it is beyond the scope of our current manuscript as there are many different combinations of ligands and complexes

along with appropriate controls which are more appropriate to be investigated in future work studying the MTR complexes.

Therefore, we have added a sentence to the discussion to stress the hypothetical nature of the proposed AF3 architectures of both the MTR-MMADHC and MTR-MTRR complexes.

Line 667-668: "Additionally, the predicted AF3 models await experimental confirmation of the specific proposed interfaces between MTR and its binding partners."

We also stress that Figure 7 is a hypothetical mechanism for MTR's function and complex formation in the discussion and we have changed the figure title accordingly.

Line 618-620: "Figure 7 integrates our cryo-EM snapshots, AF3 predictions, biophysical data, and interpretation of previous studies along with other structures, into a hypothetical composite mechanism…."

Also, we wish to point out that we have stressed in the figures and discussion that the AF3 models are predictions only.

The manuscript also makes several assignments regarding cobalamin coordination and oxidation state, particularly in distinguishing His-on versus His-off HOCbl-bound states. While the structural distinctions are convincing, the oxidation state of cobalt is inferred rather than directly measured. Given the central role of cobalt redox chemistry in MTR catalysis and reactivation, complementary spectroscopic evidence (such as UV–Vis and/or EPR) obtained on the same samples used for structural analysis would substantially strengthen these assignments. If such data are not available, the corresponding language should be framed more cautiously.

Response- Thank you for your comment. We agree with the point that we are just inferring the oxidation state of the cobalamin in our His-off and His-on structures. We do not have EPR or UV-Vis measurements readily available. Additionally, as the His-on coordination is observed in a sample that is mostly His-off it may be difficult to deconvolute their corresponding oxidation states.

Therefore, we have altered our language along with Figure 2 and Figure 4 to be cautious in our interpretation. In Figures 2 and 4 we have changed the labels from "cob(II)alamin" and "cob(III)alamin" to "cobalamin". We also have edited removing any description of our models as cob(II)alamin or cob(III)alamin and added the below sentence in the Result section

Line 248-252: "This is similar to what has been observed from studies on the C-half of the E. coli MetH protein where two crystal structures were reported: One bound to HOCbl in the His-off state (PDB 3IVA) reported to be cob(II)alamin and another bound to HOCbl in a His-on state reported to be cob(III)alamin (PDB 3IV9)29. Our His-off and His-on cryo-EM structures may adopt the same oxidation states, but further analysis is needed."

Finally, the overall study design would benefit from clearer presentation in the context of the catalytic cycle and the experimentally observed bound states in the early section of

the manuscript. In particular, it is not immediately clear how the EM structure shown in Figure 2 maps onto two cobalt oxidation states summarized in Figure 7. The authors may wish to consider adding a schematic figure that explicitly links each structural snapshot (e.g., MeTHF, OHCbl, MeCbl) to its corresponding position in the catalytic and reactivation cycles, including the associated ligand and oxidation states. Such a figure would help readers more easily follow how the cryo-EM structures, biochemical assays, and proposed mechanistic model fit together.

Thank you for the comment. In terms of the catalytic cycle, we have not directly touched on all the states involved, apart from our cryo-EM analysis of the MeCbl bound state (Figure 3) which we hypothesise to be representative of MTR after reactivation and in-between the methyl transfer from MeTHF to Hcy.

We intended that Figure 7 explicitly referred to our own work using cryo-EM and AF3 predicted structures for each state, along with other crystal structures of the bacterial homologue MetH, and all these were labelled under each cartoon state. To increase clarity, we have now changed the colour of the labelling to purple to highlight which figures correspond to which state along with previously reported crystal structures. We have also labelled each state His-on and His-off when appropriate. Specifically for the Figure 2 maps we have labelled the cartoons as Fig. 2a and Fig. 2b respectively. The Figure 7 legend has also been edited as below:

Line 647-658: “Fig. 7 | Hypothetical mechanism of cobalamin loading, reactivation, and catalysis of MTR. A schematic model showing various cartoons of proposed conformations and complexes for human MTR with MMADHC and MTRR to illustrate three major aspects of its function: (i) cobalamin loading, (ii) reactivation cycle, and (iii) catalytic cycle. Please refer to the main text for more details for each aspect. Different Cbl oxidation states are stated and represented as light orange for Cbl(II) (cob(II)alamin), red for Cbl(III) (cob(III)alamin), and dark cyan for Cbl(I) (cob(I)alamin). Domains are coloured as previously depicted in previous figures. Supporting data for each step is referred to in the purple labels such as Fig 2a cryo-EM data corresponds to the reactivation state and Fig. 2b corresponds to the reactivated transition state. PDB codes refer to previously determined structures of MetH. This hypothetical mechanism is not comprehensive. Therefore, other conformations and complexes may also occur.”

Additionally in the discussion describing this hypothetical mechanism we refer to the corresponding figure, whether its cryo-EM data or AF3 prediction, and with any previous published structures of MetH by their PDB code.

Other comments: editorial corrections and clarifications

Page 2, line 84
“MMACHC” should be corrected to MTRR.

Response- Thank you for the suggestion, however the text is meant to be MMACHC, as it is the enzyme responsible for initial cobalamin processing by removing any upper axial ligands. This subsequently allows MMADHC to bind and chaperone the processed cobalamin to MTR in the cytosol or methylmalonyl-CoA mutase in the mitochondria.

Page 2, line 90

The abbreviation "Conn" may not be standard terminology in the literature. Please consider replacing it with a more descriptive term.

Response- Thank you for the suggestion. We have replaced the abbreviation "Conn" to the full "Connecting Domain" throughout the text and Supplementary Figures 17 and 18. We also use the abbreviation "CD" in our schematic domain diagrams in Figures 5 and 6.

Page 18, line ~21
"Effect" should be replaced with "affect" where the verb form is intended.
Response- Thank you for the suggestion. It was replaced accordingly.

Page 16, line ~582 ("…explains its long hinge region")
The wording could be read as implying a mechanistic explanation without clear context. Please elaborate and clarify whether the data demonstrate or are merely consistent with the proposed mechanism.

Response- Thank you for the suggestion. We have replaced the phrase mentioned above to

Line 601-603: "<u>These observations are consistent with the long hinge region of MTRR facilitating protein–protein interactions by enabling MTR to accommodate engagement at multiple sites at the same time.</u>"

Page 16, line ~592 ("…to the lower sequence conservation")
Please clarify whether the stated differences arise from specific interacting residues, linker length, or a combination of both.'

Thank you for the suggestion. The differences in the Linker 4 sequence are on both its size and residues composition, so we have amended the text as below:

Line 614-617: "<u>This lack of cross-reactivity in reactivation could be due to the lower sequence conservation in the region of MTR that is responsible for forming this extended interaction with MTRR, with both loop length and residue composition varying depending on the organism.</u>"

Page 17, Figure 7
Panel labeling appears inconsistent: "(i) Reactivation Cycle" should be "(ii) Reactivation Cycle."

Response- Thank you for the suggestion. The label was amended in the Figure 7 as "(ii) Reactivation Cycle".

Page 18, line 655 ("insights into eukaryote-unique aspects")
This sentence would benefit from a more concise summary of the eukaryote-specific features highlighted by the study.

Response- Thank you for the suggestion. We have amended the sentence with a summary of the two important eukaryote-specific features of MTR (Cbl loading and reactivation). The phrase was changed

Line 684-687: "Importantly, we reveal insights into eukaryote-unique aspects in terms of the complexes that MTR forms to support its function, from chaperone-mediated cobalamin loading by MMADHC to multi-domain engagement by MTRR during the reactivation cycle."

Reviewer #3 (Remarks to the Author):

I co-reviewed this manuscript with one of the reviewers who provided the listed reports. This is part of the Nature Communications initiative to facilitate training in peer review and to provide appropriate recognition for Early Career Researchers who co-review manuscripts.

Response- Thank you for your review on our submitted manuscript. We appreciate the comments and have carefully considered all of them.
